# The Sex Differences in Uveal Melanoma: Potential Roles of EIF1AX, Immune Response and Redox Regulation

**DOI:** 10.3390/curroncol28040245

**Published:** 2021-07-23

**Authors:** Feng Liu-Smith, Chi-Yang Chiu, Daniel L. Johnson, Phillip Winston Miller, Evan S. Glazer, Zhaohui Wu, Matthew W. Wilson

**Affiliations:** 1 Department of Preventive Medicine, Hamilton Eye Institute, University of Tennessee Health Science Center, Memphis, TN 38105, USA; chiu@uthsc.edu; 2 Department of Dermatology, Hamilton Eye Institute, University of Tennessee Health Science Center, Memphis, TN 38105, USA; 3 Molecular Bioinformatics Core, Hamilton Eye Institute, University of Tennessee Health Science Center, Memphis, TN 38105, USA; djohn166@uthsc.edu (D.L.J.); pmille26@uthsc.edu (P.W.M.); 4 Department of Surgery, Hamilton Eye Institute, University of Tennessee Health Science Center, Memphis, TN 38105, USA; eglazer@uthsc.edu; 5 Department of Radiation Oncology, Hamilton Eye Institute, University of Tennessee Health Science Center, Memphis, TN 38105, USA; zwu6@uthsc.edu; 6 Department of Pathology, Hamilton Eye Institute, University of Tennessee Health Science Center, Memphis, TN 38105, USA; 7 Department of Ophthalmology, Hamilton Eye Institute, University of Tennessee Health Science Center, Memphis, TN 38105, USA; mwilson5@uthsc.edu

**Keywords:** uveal melanoma, sex difference, EIF1AX, redox regulation, immune response, epidemi-ology, genomics analysis

## Abstract

Background: Uveal melanoma (UVM) is a rare cancer that shows sex difference in incidence and survival, with little previous report for the underlying mechanism. Methods: This study used the SEER data (1974–2016) for an age-dependent analysis on sex difference in UVM, and further used the TCGA-UVM genomics dataset for analyzing the differential gene expression profiles in tumors from men and women. Results: Our results demonstrate a sex difference in older age (≥40 years) but not in younger patients, with men exhibiting a higher incidence rate than women. However, younger women have shown a continuous increasing trend since 1974. Examining the 11 major oncogenes and tumor suppressors in UVM revealed that EIF1AX showed a significant sex difference in mRNA accumulation and copy number variation, with female tumors expressing higher levels of EIF1AX and exhibiting more variations in copy numbers. EIF1AX mRNA levels were significantly inversely correlated with EIF1AX copy numbers in female tumors only, but not in male tumors. Differential gene expression analysis at the whole genomic level identified a set of 92 protein-coding and 16 RNA-coding genes which exhibited differential expression in men and women (fold of change cutoff at 1.7, adjusted *p* value < 0.05, FDR < 0.05). Network analysis showed significant difference in immune response and in disulfide bond formation, with EGR1/EGR2 and PDIA2 genes as regulators for immune response and disulfide bond formation, respectively. The melanocortin pathway which is linked to both melanin synthesis and obesity seems to be altered with unclear significance, as the sex difference in POMC, DCT/TYRP2, and MRAP2 was observed but with no clear direction. Conclusion: This study reveals possible mechanisms for the sex difference in tumorigenesis of UVM which has potentials for better understanding and prevention of UVM.

## 1. Introduction

Uveal melanoma (UVM) is a cancer of the eye, specifically involving three uveal melanocytic cell types: the iris, the ciliary body, and the choroid (collectively referred to as the uvea) [1]. Etiology of uveal melanoma is distinctively different from that of skin melanoma (cutaneous melanoma, CM) and mucosal melanoma despite all melanomas arising from melanocyte cells that produce melanin. Solar UV radiation (UVR) is an environmental risk factor for CM, while the UVR effect on UVM remains under debate. Nevertheless, the eye color and skin color are phenotypic risk factors for both UVM and CM. Like CM, UVM is also positively associated with a higher social economics status as indicated by area-based socioeconomic measures [2,3,4].

Epidemiology of UVM was published previously based on data from cancer registries [5]. However, there was inaccuracy of classifications due to heterogenous tumor types in some previous reports. Particularly, some studies included retinoblastoma, while other studies included other cancer types which arise from the orbital structure [6]. Although these other types of eye cancers count for ~20% of all eye cancers, there is a concern that the trend of this rare cancer is not accurately reflected.

Sex difference in UVM is documented, with men showing higher incidence rates than women in general [1,7], which is similar to that in CM [8]. We have previously found that sex difference in CM is age-dependent, with older men showing a higher incidence rate than older women while younger women show higher incidence than younger men [8,9]. The age dividing line is approximately around women’s menopause (~50 years). The age-dependent sex difference may not only reflect the behavior difference of men and women at different ages (i.e., exposure to indoor and outdoor UV radiation), it may also reflect an intrinsic difference in pathophysiological aspects of the disease etiology such as changes in the sex hormone levels. Direct evidence of involvement of sex hormone and their receptors is limited or controversial in the literature for both CM and UVM [10,11]. Therefore, the first part of this study attempts to analyze age-specific UVM incidence rates in men and women, in order to develop a hypothesis for mechanistic explanation in incidence and prognosis.

Genetic and somatic mutations are important causes for UVM [12]. Monosomy 3 (including loss of BAP1 copy number, or loss-of-function mutations in BAP1 gene) causes multiple cancer phenotypes including UVM [13]. Loss of BAP1 drives metastasis and is associated with poorer survival of UVM patients [14,15]. Somatic DNA mutations in UVM include GNAQ, GNA11, PLCB4, SF3B1, SRSF2, EIF1AX, CNKSR3, CYSLTR2, and YAP1 [16,17]. Among these genes, about 83% of UVM tumors have mutations in either GNAQ or GNA11 [18,19]. Although nearly mutually exclusive, mutations in GNAQ and GNA11 in metastatic UVM are presented at different rates, with GNA11 mutations more frequently associated with metastatic UVMs [18]. PLCB4 and CYSLTR2 mutations are usually present in UVMs that lack GNAQ or GNA11 mutations, and occur in small percentage of UVMs [20]. PLCB4 encodes a phospholipase C, while CYSLTR2 (Cysteinyl-Leukotriene Receptor 2) encodes a G protein-coupled receptor. CYSTLTR2 and PLCB4 can initiate mutations along with GNAQ and GNA11, while BAP1, EIF1AX, and SF3B1 can promote mutations. SF3B1 and SRSF2 both encode splicing factors and play key roles in the alternative splicing of mRNA, which affects cell cycle progression and cell death [21,22]. CNKSR3 amplification is associated with better survival of UVM [23]. The EIF1AX is located on the X chromosome and encodes a eukaryotic translation initiation factor 1A. Frequently mutated in a number of cancer types including carcinomas and UVM, EIF1AX is considered a novel oncogenic driver [24,25]. Molecularly, EIF1AX is essential for the assembly of 43S pre-initiation ribonucleoprotein complexes for protein synthesis [26]; a mutant form of EIF1AX was able to increase general protein synthesis in thyroid carcinoma [26], which is consistent with higher protein synthesis demand in cancer cells. The YAP1 gene is well studied for its function in promoting tumorigenesis. In uveal melanoma, YAP1 acts downstream of GNAQ/GNA11 signaling to promote cell proliferation [27]. To the best of our knowledge, there have been no reports on sex difference in the above-mentioned mutations, or systematic analysis of gene expression difference in uveal melanoma.

## 2. Materials and Methods

UVM incidence data source and analysis: US SEER18 research data (1975–2016) was downloaded using the SEERStat software (Version 8.3.8). The selection criteria for UVM followed the International Classification of Diseases for Oncology, third edition (ICD-O-3): “Primary site = C69.2, retina; C69.3, choroid; C69.4, ciliary body”, “Morphology = 8720–8790, nevi and melanomas” and “Behavior = 3, malignant”. US 2000 standard population was used for age-standardization. The annual percentage change of incidence rates was analyzed using the Joinpoint Regression Program, Version 4.8.0.1, downloaded from the SEER website. The age-standardized incidence rates were used for trend analysis. Statistical analysis was carried out by Stata IC13 software (College Station, TX, USA).

UVM genomics data and analysis: The TCGA-UVM data (mutation, copy number variation, mRNA levels normalized by RSEM algorithm [28], clinical data, and patient information) was downloaded from the GDC Data Portal (https://portal.gdc.cancer.gov/projects/TCGA-UVM, accessd on 22 July 2021) [17]. Analysis on individual gene level was carried out by Stata IC13 software. For analysis of sex differentiated gene expression at the genomic level, genes with a RSEM value of less than 1 were removed. The DESeq function (DESeq2 program) was used to determine differential expression between sexes [29]. All genes that failed to yield a *p* value less than 0.05 and a fold change greater than 1.7 were removed. The Benjamini–Hochberg false discovery rate procedure was performed on the trimmed gene list [30]. All genes that failed to yield a false discovery rate of less than 0.05 were removed. Significant protein-coding genes were then uploaded to the STRING v11 website for functional protein association network analysis [31]. Significantly enriched pathways and annotated keywords were defined by Benjamini–Hochberg procedure adjusted *p* values (i.e., false-discovery rate) of less than 0.05.

## 3. Results

### 3.1. The Sex Difference Analyzed by Epidemiological Methods

#### 3.1.1. The Age-Dependent Sex Disparity in Uveal Melanoma from the SEER Dataset

The uveal melanoma data was downloaded from SEER 18 registries with SEERStat software (Version 8.3.8), which included all cases from 1975 to 2016. A total of 5097 female and 5576 male cases were included according to our selection criteria (see Method section). The mean diagnosis age was 60.8 ± 14.1 years (mean ± sd). The mean diagnosis age for men and women was 60.5 ± 13.9 and 61.1 ± 14.3, respectively, exhibiting a small but significant difference (*p* = 0.027, Student’s *t*-test), with men diagnosed at a slightly younger age.

As shown in Table 1, analysis of age-specific incidence rates revealed that women showed a non-significant higher incidence rate at a younger age (<40 years), while men showed a significant higher incidence rate at older ages (≥40 years). Most uveal melanomas are diagnosed at an older age: only 8.2% of cases were diagnosed at ages under 40, 91.8% of cases were diagnosed at age 40 and older. When the age category was divided into two groups (<40 and ≥40 years), the age-standardized incidence rate was 0.32 and 0.34 per million for younger females and males (*p* = 0.334, one-sided), and 4.54 and 6.18 per million for older females and males (*p* < 0.0001, one-sided), respectively. Of all ages, women showed an incidence rate of 2.14 per million, while men showed an incidence rate of 2.86 per million person-years (age-adjusted, *p* < 0.0001, two-sided). Even though men showed a significant overall higher incidence of UVM, this was only observed in the older ages. There was no significant sex disparity of UVM at younger age.

#### 3.1.2. The Trend of UVM Incidence Rates over Years

**Figure 1:**
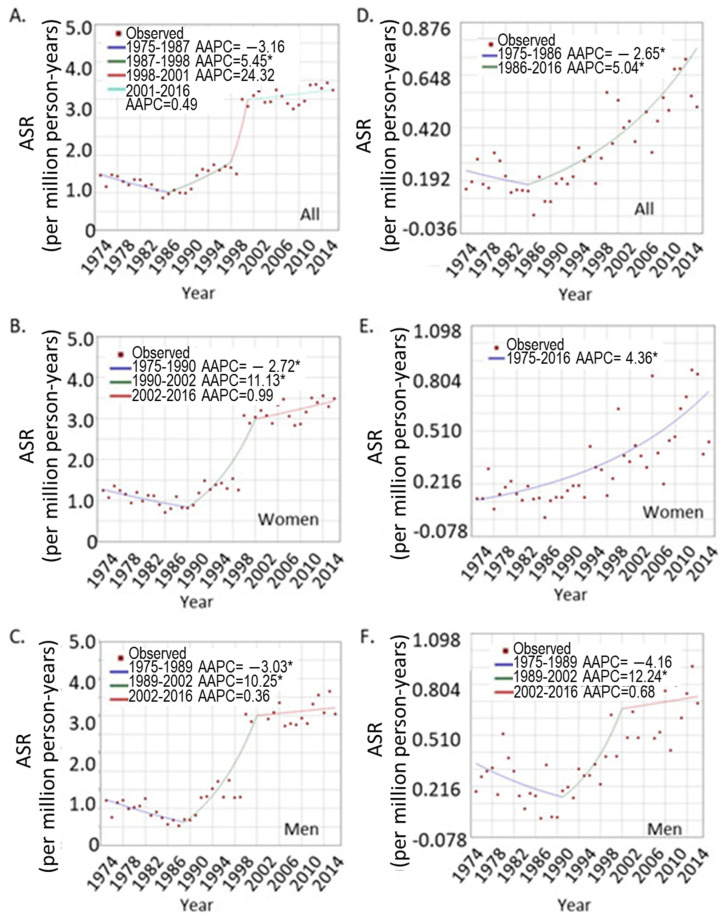
Joinpoint analysis of the UVM trends over the years. (**A**–**C**), overall trend for UVM at all ages; (**A**), both sexes; (**B**), women; and (**C**), men. (**D**–**F**), trends for younger patients under age 40; (**D**), both sexes; (**E**), women, (**F**), men. * indicates significant trend of *p* < 0.05.

In order to track the trend of UVM incidence rates, the age-standardized incidence rates were calculated based on US 2000 standard population for each year. The incidence trend was analyzed using Joinpoint Regression Program (Version 4.8.0.1). As shown in Figure 1, UVM exhibited an increasing trend from 1975 to 2016, with an average annual percentage change (AAPC) of 2.6 (95% CI, 0.3, 4.9, *p* < 0.0001). A final model of three points (three segments of changing patterns) was selected by the program upon 4500 permutation tests, which revealed an overall decreasing trend from 1975 to 1987 (AAPC of −3.2%, 95% CI −4.9, −1.4). A significant increasing trend was observed from 1987 to 1998 (AAPC of 5.4, 95% CI 3.1, 7.9). There was a sharp increase from 1998 to 2001 (AAPC of 24.3, 95% CI −1.6, 57.2), but this did not reach significance level (*p* = 0.10, Figure 1A). No significant changes were observed from 2001 to 2016. When sex was considered separately, the trend was similar in men and women, both of which were similar to the overall trend, i.e., a decrease until the late 1980s which was followed by a significant increase from the late 1980s to early 2000s. After that, the rates stayed stable in both sexes (Figure 1B,C).

#### 3.1.3. The UVM Incidence Trend in Different Age Groups

In order to determine the age-specific incidence trend, the UVM patients were grouped into younger (<40 years of age) and older age groups (≥40 years), and age-standardized rates were calculated for each group. The incidence trend was analyzed as above. As shown in Figure 1D, when both sexes were combined, there was a significant increasing trend in the younger age group from 1986 to 2016 (AAPC = 5.04, 95% CI 3.5, 6.6). A sex-specific difference in trend was observed in the younger age group. Younger women showed a continuous increasing incidence from 1975 to 2016 (Figure 1E). Joinpoint analysis did not divide the years into different segments, with an AAPC of 4.4% (95% CI 3.2, 5.5). In contrast, younger men showed a pattern similar to the entire group: a decrease at the beginning and then an increase, and then a maintained flat line. The AAPC for younger males was a nonsignificant 1.7% (95% CI −1.3, 4.7, Figure 1F). However, the trends for men and women were similar in the older age group.

### 3.2. The Sex Difference in Tumor Genomic Analysis

#### 3.2.1. The Sex Difference in Major Oncogenes from the TCGA UVM Patients: Higher EIF1AX Expression in Female Tumors

In order to determine where there is major difference in mutation burdens in tumors derived from male and female patients, we examined the mutation rate, copy number variation, and mRNA expression levels of the major oncogenes or tumor suppressors including GNAQ, GNA11, BAP1, PLCB4, SF3B1, SRSF2, EIF1AX, TERT, CNKSR3, CYSLTR2, and YAP1. The TCGA-UVM level 3 data contained point mutations, copy number variations and mRNA RSEM data which were all included in our analysis. Among these 11 genes, there was no significant sex difference in frequencies of mutations (including point mutation and copy number variation) in GNAQ, GNA11, BAP1, and SF3B1 genes in UVM. No mutation was found for TERT and CNKSR3 genes, and other genes showed small numbers of mutations (three for SRSF2, three for CYSLTR2, two for PLCB4, and one for YAP1), not suitable for statistical comparison between sexes. A total of 10 EIF1AX mutations were identified (6 out of 35 female tumors and 4 of the 45 male tumors); thus, there is no sex difference in the EIF1AX mutation frequency (*p* = 0.27, χ^2^ test).

We determined no sex difference in copy number variation for BAP1, SF3B1, CYSLTR2, PLCB4, TERT, CNKSK3, and YAP1 genes. Seven female tumors and five male tumors gained one copy of EIF1AX, while another seven female and three male tumors lost one copy of the EIF1AX gene (Table 2). The altered copy number (loss and gain combined) is more frequent in female tumors (*p* = 0.027, χ^2^ test). None of the EIF1AX mutants showed any copy number variation.

At mRNA level among the above-mentioned oncogenes/tumor suppressors, EIF1AX was the only gene that showed a significant sex difference, with female tumors expressing higher levels of mRNA (Table 2). The mean mRNA level for females was 1108.6 ± 101.0 (mean ± standard error) RSEM estimate, while the mean for male tumors was 841.7 ± 65.9 (*p* = 0.024, Student’s *t*-test, unadjusted). Since EIF1AX is located on the X chromosome, we selected two X-located genes flanking EIF1AX (EGFL6, EIF2S3) as control genes to examine whether incomplete X inactivation caused the differential expression of EIF1AX.

The control genes showed no sex difference in UVM tumors at mRNA level (Table 2). Since EIF1AX also showed sex-differentiated copy number variation, the correlation between copy number variation and mRNA RSEM levels were analyzed by a linear regression model. Interestingly, the copy number of EIF1AX was significantly inversely correlated with EIF1AX mRNA (*p* = 0.030), with tumors which lost one copy of EIF1AX (EIF1AX_-1) expressing higher levels of mRNA (Table 2). When the linear regression was performed in male and female tumors separately, the correlation was significant only in female tumors (*p* = 0.047) and not in male tumors (*p* = 0.35) (Table 2).

There is no significant difference in mRNA levels in the EIF1AX wild-type and mutant tumors, however the mutations play critical roles in tumorigenesis. Thus, we repeated the above linear regression model with the exclusion of patients carrying the EIF1AX mutations (*n* = 10). The results were similar, with an overall significant inversed correlation (including both men and women, *p* = 0.034). Women showed a borderline significant correlation (*p* = 0.054), while men did not show a significant correlation (*p* = 0.32) (Table 2).

#### 3.2.2. The Global Sex-Differentiated Gene Expression Profile in UVM Tumors

In order to obtain a comprehensive understanding of the sex difference in UVM, the TCGA-UVM dataset was used for differential gene expression analysis. All 80 tumors were included (45 men and 35 women). RSEM normalized counts and DeSeq2 software were used for differential gene expression analysis between men and women. The cutoff *p* value and FDR (false discovery rate) were both set at 0.05. A total of 92 protein-coding genes exhibited differential expression in men and women (fold of change cutoff at 1.7), among which nine were located on the Y chromosome and had no expression in tumors from female patients (except for one gene, RPS4Y, which had detectable low expression in two tumors from two female patients). Additionally, 16 RNA-coding genes (including pseudogenes) showed differential expression in tumors from two sexes (fold of change cutoff: 1.7, Supplemental Table S1). The top four overexpressed genes in male-derived tumors were IGK (immunoglobulin kappa constant), IGLL5 (immunoglobulin lambda-like polypeptide 5), CD79A (CD79a antigen, B-Cell antigen receptor complex-associated protein alpha chain), and JCHAIN (joining chain of multimeric IgA and IgM), all related to immune function. The top five over-expressed genes in female tumors were RBM24 (RNA binding motif protein 24), PDIA2 (protein disulfide isomerase family A member 2), SCARA5 (scavenger receptor class A member 5) and DCT (dopachrome tautomerase). The RBM24 gene is responsible for alternative splicing while the PDIA2 is a gene catalyzing thiol-disulfide interchange reactions and also modulating estradiol activity through direct binding. SCARA5 is a ferritin receptor mediating non-transferrin-dependent iron transfer. DCT is a key enzyme in melanin biosynthesis and detoxification of melanin intermediates [32]. Lower DCT activity (*slaty* mutation) in mice is associated with a switch of melanin synthesis from protective eumelanin to deleterious pheomelanin [32]. A second gene with a function in melanin signaling, POMC (proopiomelanocortin) showed a 3.0 fold rate of up-regulation in male tumors (Supplemental Table S1). A third gene that is relevant to melanin metabolism is MRAP2 (melanocortin 2 receptor accessory protein 2), which was down-regulated in male tumors (–2.6 fold, Supplemental Table S1).

The 92 proteins were subjected to STRING analysis [31], however only 82 were recognized by the program. All Y chromosome-located proteins were excluded by the program. The IGK gene was also excluded, which has an apparent immune function. This may be because this locus encodes a number of rearranged immunoglobin light chains, therefore, not being recognized as a single protein. The results showed that no significant molecular function (Gene Ontology, GO) was identified. In the cellular component GO category, all significant GO groups were related to immune response which includes immunoglobin complexes and cell membrane/cell surface components (Supplemental Table S2). No significant KEGG pathway or reactome pathway was identified. The UniProt annotated keyword analysis returned keywords “Glycoprotein” (*p* = 1.65 × 10^−6^), “Disulfide bond” (*p* = 1.65 × 10^−6^), and ”Signal” (*p* = 6.4 × 10^−5^) (Supplemental Table S2). A total of 42, 36, and 32 of the 82 genes were included in the Glycoprotein, Disulfide bond, and Signal groups, respectively, all with very low *p* values. Protein domain analysis returned “Early Growth Response, N-terminal” which included EGR1 and EGR2 genes (*p* = 0.05), both of which were down-regulated in tumors from men as compared to female tumors.

## 4. Discussion

The age-dependent sex difference in UVM is summarized by analyzing the SEER data. As reported before, men have a higher incidence of UVM than women, but this difference was caused by the disparity in older age only. At younger age (<40 years) there was no sex difference, unlike the cutaneous melanoma which showed substantial difference at both young and older age [9]. Over the years, UVM showed a sharp increase from year 1990 to 2000, and then maintained a slow and non-significant increasing trend. A more comprehensive reporting system for UVM cases may be the reason for the increase in reported cases, as many cases were diagnosed outside of cancer centers and may not have been registered (clinical observation by Dr. Mathew Wilson, also [33]). The younger age group, though, showed significant increase from 1986 to 2016. This trend is especially obvious in young women but not in young men (Figure 1E,F). The higher cancer incidence in older men is a common phenomenon if all cancer sites are taken into consideration [34]. The attributing factors are not quite clear, but may be related to both pathophysiological changes (intrinsic changes following aging) and behavior difference in the two sexes; for example, smoking and drinking is more prevalent in men. The intrinsic sex hormone changes may also play an important role, as sex hormones regulate essentially all aspects of cellular activities, which include immune responses, oxidative regulation, and even DNA repair [35]. Overall, the underlying driving force warrants further investigation.

EIF1AX was identified to express at significant higher levels in female tumors than in male tumors, and it also exhibited an unusual correlation with copy number. A comprehensive study showed that while most genes showed a positive correlation between mRNA level and copy numbers, about 1% of genes showed inversed correlation, i.e., higher copy number was associated with lower mRNA levels [36]. It is unclear how this gene is regulated; however, it is clear that female tumors showed higher levels of mRNA. A common variant of EIF1AX (A113_splice mutation) found in thyroid cancer is often associated with the RAS oncogene and drives thyroid cancer development [26]. Thyroid cancer incidence is about three times higher in women than in men [37], and perhaps EIF1AX plays a role. In UVM, however, further investigation is needed to validate and explain why female tumors express higher levels of EIF1AX. If higher EIF1AX mRNA is a driving force in women, then it may explain why women survive better than men as EIF1AX is an indicator for Class 1 GEP (gene expression profiling) tumors which usually show better overall survival [38].

Our STRING network analysis using 82 out of the 93 protein-encoding genes revealed significant GO cellular component functions in interlinked immunoglobin and cell surface/plasma membrane GO components. This is cross-validated by the annotated keyword analysis which revealed that 51.2% (42/82) of the genes in the gene set encode proteins that can be glycosylated. A major function of glycoproteins is their involvement in immune response. These results, therefore, strongly suggest that the sex difference in UVM is perhaps due to differential immune responses in men and women. Over-expression of the IGK, IGLL5, CD79a, and JCHAIN in males also supports that men may show a more inflammatory microenvironment than women, and thus provoke more immune responses to deal with it. This is perhaps due to a more rapid resolution of inflammation in women than in men in general [39]. Furthermore, the significant protein domain “Early Growth Response” includes EGR1 and EGR2 genes, which are transcriptional factors controlling the TCR-mediated differentiation of natural killer T cells [40]. Both EGR1 and EGR2 are down-regulated in male tumors, indicating possible fewer NKT cells infiltrated in the tumors from men. These various lines of evidence all point to a more inflammatory microenvironment and a less efficient immune system in men, which may provide a possible molecular mechanistic explanation for the sex disparity in UVM.

Another network analysis that is cross-validated by differential gene expression and annotated keyword analysis is the redox-linked disulfide bond. The PDIA2 (PDIp, PDA2) gene belongs to the PDI gene family, which belongs to a larger redox thioredoxin gene family [41]. PDI enzymes catalyze thiol-disulfide exchange reactions to maintain the correct protein folding and activities; additionally, the disulfide bonds can be formed abnormally under oxidative stress. Men usually exhibit a higher level of oxidative stress than women [42], suggesting higher levels of oxidation of thiol groups, and requiring more PDI enzymes. However, male tumors showed 4.5-fold lower PDIA2 levels than female tumors (Supplemental Table S1), which suggests a poorer capacity to cope with oxidative stress. In pancreatic tissue, the PDIA2 targets pancreatic digestive enzymes and prevents formation of inactive aggregates [43]. The PDIA2 protein is an endoplasmic reticulum-located glycoprotein [44,45], exhibiting high affinity with estrogen and serving as a possible intracellular estrogen regulator in vitro and in vivo [46]. Thus, it is not a surprise that PDIA2 is down-regulated in male tumors as compared to female tumors, as it is expected that female cells may use this enzyme as a local estrogen regulator. These results are consistent with reports that estrogen helps to deal with oxidative stress in women [47]. Additionally, PDIA2 can directly bind to the human major histocompatibility complex class 1 antigens (HLA-A, B, and C) and play a role in antigen presentation [48]. Taken together, with the multifunction of PDIA2 in cells, it is likely that the differential expression of this gene provides an important layer of mechanistic explanation for how sex hormones are linked to immunity regulation as well as redox regulation, both of which exhibit substantial difference in men and women.

Another characteristic of uveal melanoma is the production of melanin pigment [49]. The TCGA-UVM data confirmed that MC1R, MC4R, and MC5R were expressed in uveal melanoma, with MC1R exhibiting the highest expression level (data not shown). The significant sex difference shown in the expression of DCT and POMC was unexpected, with POMC up (3.0 fold) and DCT down (−3.8 fold) in male tumors. POMC gene products include α-MSH, β-MSH, and β-endorphin, playing roles in pain-sensing, pigment synthesis, and immune modulation. The α-MSH peptide binds to MC1R and other receptors to stimulate pigment synthesis and regulate immune responses [50]. Men showed an average higher level of plasma α-MSH than women [51], and it is known that human melanocytes can produce local α-MSH [52]. The role of MRAP2 in melanin signaling is unclear, but loss-of-function MRAP2 variants are associated with obesity [53], which also involves α-MSH, Mc1R, and MC4R signaling. Overall, it is unclear how the shared melanin and obesity signaling plays roles in UVM. These pathways warrant further investigation.

A major limitation of the genomic data analysis is the small sample size—a total of 80 tumors in the TCGA-UVM dataset. Thus, whether the above-mentioned pathways are indeed reflecting true sex difference needs validation from a larger cohort. Another major limitation of this study remains the population-wide and associative nature of the studies. We have identified two novel mechanisms in which UVM is potentially stimulated, which may be ultimately caused by variations in sex hormone levels. The complex nature of sex hormone biology is challenging to investigate in this study. Detailed molecular studies at cellular level are required to validate the genomics findings. In addition, given the rarity of UVM and the retrospective nature, epidemiological analysis is limited by available data and variables therein.

## 5. Conclusions

In summary, from the sex differentiated UVM risk and genomics analysis, there is a need to investigate the cause of UVM in both sexes. This study suggests that immune responses and redox regulations may play important roles in UVM etiology, which, upon validation, can be used as prevention and therapeutic targets.

## Figures and Tables

**Table 1:** Age-specific incidence rate difference in uveal melanoma.

	Case Number			IR (Per Million) *		IRR	*p* Value
Age Category	Female	Male	Total	Female	Male	F/M	One-Sided
01–04 years	1	0	1	0.007	0.000	n/a	n/a
05–09 years	0	3	3	0.000	0.019	n/a	n/a
10–14 years	2	8	10	0.013	0.049	0.26	0.0380
15–19 years	28	22	50	0.174	0.130	1.34	0.1540
20–24 years	46	39	85	0.276	0.225	1.23	0.1730
25–29 years	77	66	143	0.45	0.38	1.19	0.1550
30–34 years	122	120	242	0.72	0.70	1.02	0.4450
35–39 years	142	185	327	0.88	1.15	0.76	0.0064
40–44 years	223	282	505	1.45	1.88	0.77	**0.0019**
45–49 years	327	412	739	2.29	2.99	0.77	**0.0002**
50–54 years	495	577	1072	3.72	4.58	0.81	**0.0004**
55–59 years	601	743	1344	5.04	6.74	0.75	**0.0000**
60–64 years	587	652	1239	5.64	7.02	0.80	**0.0001**
65–69 years	693	774	1467	7.72	10.26	0.75	**0.0000**
70–74 years	578	597	1175	7.79	10.38	0.75	**0.0000**
75–79 years	484	518	1002	8.08	12.49	0.65	**0.0000**
80–84 years	372	336	708	8.47	12.85	0.66	**0.0000**
85+ years	254	191	445	6.00	10.14	0.59	**0.0000**
Total	5032	5525	10,557	2.14	2.86	0.87	**0.0000**

* Age-adjusted incidence rate. **Bolded *p* values**: significant after Bonferroni adjustments.

**Table 2:** Sex difference in EIF1AX mean mRNA levels and their inverse correlation with copy numbers of EIF1AX.

Gene and Status	All			Female			Male			*p* Value *
	N	Mean	Std. Err	N	Mean	Std. Err	N	Mean	Std. Err	
EIF1AX_all	80	958.5	59.2	35	1108.6	101	45	841.7	65.9	0.024
EFNB1_all	80	322.1	9.8	35	315.6	14.9	45	327.2	13.1	0.56
EIF2S3_all	80	7033.7	342.5	35	7546.3	570.6	45	6635.1	412.9	0.19
EIF1AX_-1	12	1271.0	227.2	7	1562.7	96	5	862.6	54.9	0.13
EIF1AX_0	58	921.3	57.8	21	1015.5	47.5	37	867.9	49.7	0.22
EIF1AX_1	10	798.8	176.3	7	933.6	68.9	3	484.3	24.1	0.27
Regression **	p1 = 0.030; p2 = 0.034			p1 = 0.047; p2= 0.054			p1 = 0.35; p2 = 0.32			

* *p* value refers to the mean comparison between sexes calculated by Student’s *t*-test, two-sided. ** Regression *p* values are estimated for the correlation between CNV and mRNA by linear regression model. EIF1AX_-1, EIF1AX_0 and EIF1AX_1 represent tumors with loss of one copy, diploid and gain of one copy for the EIF1AX gene. *p* values: p1, regression with all EIF1AX (wild-type or mutant); p2, regression with only EIF1AX wild-type genotype (excluding the EIF1AX mutants).

## Data Availability

All data is publicly available.

## Supplementary Materials

The following are available online at https://www.mdpi.com/article/10.3390/curroncol28040245/s1, Table S1: Differentiated gene expression in tumors from men and women; Table S2: Network analysis of the significant protein-coding genes listed in Supplemental Table S1.
